# An Incidental Solitary Plasmacytoma of Bone Mimicking Neuroendocrine Tumor Metastasis on 68Ga-DOTATATE Positron Emission Tomography/Computed Tomography

**DOI:** 10.4274/mirt.93064

**Published:** 2016-09-29

**Authors:** Duygu Has Şimşek, Serkan Kuyumcu, Bilge Bilgiç, Emine Göknur Işık, Cüneyt Türkmen, Işık Adalet

**Affiliations:** 1 Tokat State Hospital, Clinic of Nuclear Medicine, Tokat, Turkey; 2 İstanbul University İstanbul Faculty of Medicine, Department of Nuclear Medicine, İstanbul, Turkey; 3 İstanbul University İstanbul Faculty of Medicine, Department of Pathology, İstanbul, Turkey; 4 Hitit University, Çorum Training and Research Hospital, Department of Nuclear Medicine, Çorum, Turkey

**Keywords:** 68Ga-octreotide, DOTA(0)-Tyr(3)-, positron emission tomography/computed tomography, plasmacytoma, neuroendocrine tumors

## Abstract

A 54-year-old woman with suspicion of neuroendocrine tumor (NET) was referred for ^68^Ga-DOTATATE positron emission tomography/computed tomography (CT) imaging due to clinical findings. A well-defined osteolytic lesion on the corpus of the third lumbar vertebra was evident on CT images with mild uptake of ^68^Ga-DOTATATE, which led to suspicion of NET metastasis. Histopathologic examination revealed solitary plasmacytoma of the bone. The patient received local external radiotherapy for plasmacytoma. This case indicatesthat other diseases expressing somatostatin receptors may be inaccurately reported as tumor recurrence and highlights the importance of meticulous evaluation of positive findings.

## Abstract (tr)

Nöroendokrin tümör (NET) şüphesi bulunan 54 yaşındaki kadın hastaya ^68^Ga-DOTATATE pozitron emisyon tomografisi/bilgisayarlı tomografi (BT) görüntüleme yapıldı. Lomber 3. vertebrada BT görüntülerinde tanımlanan osteolitik lezyonda düşük düzeyde ^68^Ga-DOTATATE tutulumu izlenmesi nedeniyle NET metastazı açısından şüpheli bulundu. Histopatolojik örnekleme soliter kemik plazmasitomu olarak sonuçlandı. Hasta plazmasitoma nedeniyle lokal eksternal radyoterapiye yönlendirildi. Bu olgu somatostatin reseptörlerini eksprese eden diğer hastalıkların tümör nüksü olarak yanlış raporlanabileceğini ve pozitif bulguların dikkatli değerlendirilmesinin önemini vurgulamaktadır.

## INTRODUCTION

The primary indication of ^68^Ga-DOTA-conjugated peptide positron emission tomography/computed tomography (PET/CT) is neuroendocrine tumor (NET) imaging (1). However, tumors that express somatostatin (SST) receptors other than NETs can also be visualized by ^68^Ga-DOTA-conjugated peptide PET/CT (2). In vitro studies with plasma cell lines have shown that the SST is expressed on malignant plasma cells (3). In our case, a solitary bone plasmacytoma (SBP) in the lumbar spine showed increased ^68^Ga-DOTATATE uptake mimicking bone metastasis in a patient with suspected NET recurrence.

SBP is characterized by a solitary bone lesion that shows infiltration by plasma cells without evidence of anemia, hypercalcemia, or renal involvement suggesting systemic myeloma (4). SBP may involve any bone but most often affects the axial skeleton, particularly the vertebra, pelvis, ribs and pectoral girdle (4).

## CASE REPORT

**Figure 1:**
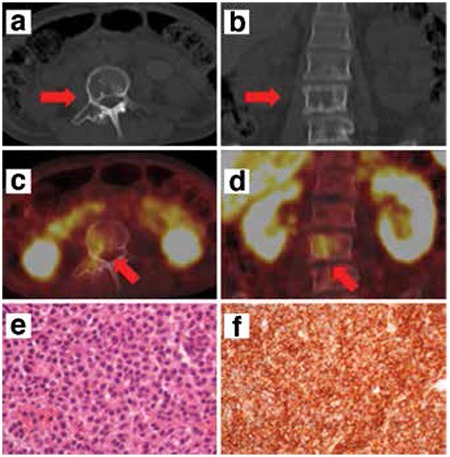
Transaxial (a)-coronal (b) computed tomography images; transaxial (c)- coronal (d) positron emission tomography/computed tomography fusion images; diffuse neoplastic plasma cell infiltration in the bone marrow (e) and CD38 antibody positivity on the cell membrane (f) in immunohistochemical and histopathologic examinations. A well- defined osteolytic lesion on the corpus of the third lumbar vertebra extending to the right pedicle (a, b arrows) showing mild ^68^Ga-DOTATATE uptake (c, d arrows)

A 54-year-old woman with suspicion of NET was referred for ^68^Ga-DOTATATE PET/CT due to clinical findings. A well-defined osteolytic lesion on the corpus of the third lumbar vertebra extending to the right pedicle was evident on CT images (Figure 1a, b; arrows). The corresponding PET images (Figure 1c, d; arrows) demonstrated mild uptake of ^68^Ga-DOTATATE, which led to suspicion of NET metastasis.

Histopathologic evaluation of the lesion was recommended in order to differentiate bone metastasis of NET from other SST expressing pathologies. Histopathologic examination demonstrated diffuse neoplastic plasma cell infiltration in the bone marrow (Figure 1e). Immunohistochemical staining revealed immunoglobulin λ-light chain antibodies in the tumor, and CD38 antibody positivity on the cell membrane (Figure 1f). All findings indicated SBP with supporting clinical data. The patient received local external radiotherapy for plasmacytoma.

## LITERATURE REVIEW AND DISCUSSION

^68^Ga-DOTA-conjugated peptide PET/CT is the imaging modality of choice for NETs for the detection of metastatic disease or local relapse, and it affects therapeutic approach in more than 40% of patients (5,6,7). The most common sites of NET metastasis are the liver, lymph nodes and bone (8). The presence of bone metastasis has vital clinical importance on treatment management, since it has been shown that bone metastasis is associated with poor overall survival (6).

Although results of ^68^Ga-DOTA-conjugated peptide PET/CT in NETs are remarkable other tumors that express SST (predominantly SST2, SST3 and SST5), such as lymphomas, breast and lung cancers, thyroid cancers, gastrointestinal stromal tumors, prostate cancers and plasmacytoma/multiple myelomas, can also be avid for ^68^Ga-DOTA-conjugated peptide PET/CT, thus misleading the physician (2).

It is not unusual that SBP has avidity of ^68^Ga-DOTATATE. Previous studies have shown that ^111^In-pentetreotide SST scintigraphy is an alternative method to displayin vivo multiple myeloma/SBP activity, especially in patients with relapsing disease and a more aggressive type of myeloma (9). In our case, a solitary osteolytic vertebral lesion with mild ^68^Ga-DOTATATE uptake is less likely to be a metastasis because skeletal lesions of NETs are mostly osteosclerotic. The metastasis due to NETs are osteolytic only in 10% of the cases (10). A histopathologic evaluation was required for the definite diagnosis of the bone lesion and the patient was diagnosed with SBP, not relapse.

Degenerative diseases in the spine can also lead to increased ^68^Ga-DOTATATE uptake. Klinaki et al. (11) reported a case with Modic changes in L4-5 vertebras that have caused ^68^Ga-DOTATATE uptake probably due to increased blood supply or infiltration with activated lymphocytes. Putzer et al. (12) reported a false positive lesion caused by extensive vertebral osteophytes with an inflammatory component.

In the literature, there aretwo case reports describing ^68^Ga-DOTATATE avid vertebral hemangiomas (13,14). The characteristic pattern in CT may help in distinguishing vertebral hemangioma and bone metastasis. A fibrous dysplasia of the bone also demonstrated significant ^68^Ga-DOTATATE uptake as reported by Kuyumcu et al. (15).

^68^Ga-DOTATATE has significant clinical impact that direct patients either to surgery or to systemic/palliative therapy. Thus, physicians should be careful when evaluating any lesion. Multiple bone lesions may be mistaken as metastases, and solitary lesions may reveal other diagnoses.

This case indicates that other diseases expressing SST receptors may be inaccurately reported as tumor metastasis and highlights the importance of meticulous evaluation of positive findings.

### Ethics

Informed Consent: Consent form was filled out by all participants.

Peer-review: Externally peer-reviewed.

Financial Disclosure: The authors declared that this study has received no financial support.
